# Metagenomic analysis of DNA viruses from posttransplant lymphoproliferative disorders

**DOI:** 10.1002/cam4.1985

**Published:** 2019-01-29

**Authors:** Vikas R. Dharnidharka, Marianna B. Ruzinova, Chun‐Cheng Chen, Priyanka Parameswaran, Harry O'Gorman, Charles W. Goss, Hongjie Gu, Gregory A. Storch, Kristine Wylie

**Affiliations:** ^1^ Division of Pediatric Nephrology Washington University School of Medicine St Louis MO USA; ^2^ Department of Pathology and Immunology Washington University School of Medicine St Louis MO USA; ^3^ Department of Surgery Washington University School of Medicine St Louis MO USA; ^4^ Department of Biostatistics Washington University School of Medicine St Louis MO USA; ^5^ Division of Pediatric Infectious Diseases Washington University School of Medicine St Louis MO USA; ^6^ McDonnell Genome Institute Washington University School of Medicine St Louis MO USA

**Keywords:** anellovirus, Epstein-Barr virus, lymphoma, posttransplant lymphoproliferative disorders, viral infection

## Abstract

Posttransplant lymphoproliferative disorders (PTLDs), 50%‐80% of which are strongly associated with Epstein‐Barr virus (EBV), carry a high morbidity and mortality. Most clinical/epidemiological/tumor characteristics do not consistently associate with worse patient survival, so our aim was to identify if other viral genomic characteristics associated better with survival. We extracted DNA from stored paraffin‐embedded PTLD tissues at our center, identified viral sequences by metagenomic shotgun sequencing (MSS), and analyzed the data in relation to clinical outcomes. Our study population comprised 69 PTLD tissue samples collected between 1991 and 2015 from 60 subjects. Nucleotide sequences from at least one virus were detected by MSS in 86% (59/69) of the tissues (EBV in 61%, anelloviruses 52%, gammapapillomaviruses 14%, CMV 7%, and HSV in 3%). No viruses were present in higher proportion in EBV‐negative PTLD (compared to EBV‐positive PTLD). In univariable analysis, death within 5 years of PTLD diagnosis was associated with anellovirus (*P* = 0.037) and gammapapillomavirus (*P* = 0.036) detection by MSS, higher tissue qPCR levels of the predominant human anellovirus species torque teno virus (TTV;* P* = 0.016), T cell type PTLD, liver, brain or bone marrow location. In multivariable analyses, T cell PTLD (*P* = 0.006) and TTV PCR level (*P* = 0.012) remained significant. In EBV‐positive PTLD,*EBNA‐LP*,*EBNA1* and *EBNA3C* had significantly higher levels of nonsynonymous gene variants compared to the other EBV genes. Multiple viruses are detectable in PTLD tissues by MSS. Anellovirus positivity, not EBV positivity,was associated with worse patient survival in our series. Confirmation and extension of this work in larger multicenter studies is desirable.

## 1 BACKGROUND

Posttransplant lymphoproliferative disorders (PTLDs), an abnormal proliferation of lymphoid cells under posttransplant immunosuppression, are a major malignant complication of organ and tissue transplant.^1^, ^2^, ^3^, ^4^ PTLDs have a high morbidity and 5‐year mortality that exceeds 50%.^3^ About 50%‐80% of PTLD cases are strongly related to the oncogenic Epstein‐Barr virus (EBV).^5^ Cytomegalovirus seromismatch has been associated in some studies^6^, ^7^ but not consistently. It is not known whether other viruses are also associated with PTLD. While recent mortality rates have decreased with general medical advances and newer therapies,^8^, ^9^ mortality remains high^10^ and graft failure is a significant complication of interventions.^11^ Though many prognostic indices have been used to predict survival after PTLD, mortality after PTLD is not fully explained by these indices.^12^, ^13^, ^14^ These indices vary considerably in their component prognostic factors; they do not consistently include the same clinical, viral, epidemiologic or tumor characteristics. Therefore, host responses to EBV and the degree of overall immunosuppression have been studied as possible contributors to prognosis and outcome, but still do not fully explain the outcomes.^15^, ^16^, ^17^

Our aim was to determine whether there were DNA viruses associated with EBV‐negative PTLD or PTLD outcomes using metagenomic shotgun sequencing (MSS) of archived formalin‐fixed paraffin‐embedded (FFPE) tissue samples from PTLD patients. MSS is an approach that assesses genomic material from host and microbes within a sample, allowing for the culture‐independent detection of microbes without a priori knowledge of which viral groups are present with a sample. Like genome‐wide association tools, MSS is a powerful tool for studying viruses in clinical samples because it allows evaluation of a comprehensive set of viruses simultaneously. In addition, MSS can provide genomic data that can be used to assess features or variants that may associate with virulence or pathogenicity. Our approach included the use of ViroCap^™^, a targeted sequence capture method that we developed recently to enhance detection of viral sequences by MSS.^18^ This improved methodology allows us to thoroughly characterize the viruses associated with PTLD, and eventually study how EBV genome variants contribute to more severe presentations or worse outcomes. We can also study which viruses, if any, are associated with EBV‐negative PTLD. We undertook this genomic analysis because these newer technologies could lead to new information and insights not found with other methods.

## 2 METHODS

This study was approved the Human Subjects Research Protection Office at Washington University School of Medicine.

### 2.1 Tissue sample identification

We first identified through a search of our electronic medical records that all tissue blocks from PTLD cases available in the tissue archives of the Washington University School of Medicine Pathology Department. Tissue specimens of sufficient quantity (as evaluated by hematopathologist MBR) were selected for nucleic acid extraction. We also identified 8 EBV‐negative control tissues (abdominal lymph nodes from cases of diverticulitis, prolapse, appendicitis or gunshot wound) and four positive EBV‐positive control tissues that were either Hodgkin lymphoma or diffuse large B cell lymphoma (DLBCL) from immunocompetent patients in a nontransplant setting. Pathological and clinical covariates extracted for the PTLD specimens are described in Supplementary Methods.

### 2.2 DNA extraction and MSS

DNA extraction methods are described in Supplementary Methods. We generated dual‐indexed sequencing libraries from the DNA using the KAPA Low Throughput Library Construction Kit (KAPA Biosystems, Wilmington, Massachusetts). We pooled libraries and mixed them with the ViroCap^™^ targeted sequence capture probes (synthesized by Nimblegen^®^), which target and enrich genomes from a comprehensive set of vertebrate viruses to enhance sensitivity. Targeted sequence capture was carried out according to the manufacturer's instructions. We sequenced the enriched viral nucleic acids using the Illumina HiSeq 2000/2500 platform. We analyzed sequences using a pipeline adapted from the method previously described by us^19^ except that Burroughs Wheeler Alignment tool BWA MEM was used for the nucleotide sequence alignments.^20^ To avoid false positives resulting from index swapping during capture,^21^, ^22^, ^23^ in which the library‐specific indexes are transferred between libraries at a low frequency, we subtracted 0.1% of the total viral reads for each virus within a pool from each sample. This threshold was based on published studies^21^, ^22^, ^23^ and our experience with capture of dual‐indexed sequencing libraries.^18^ Samples with viral signal above or below that threshold were considered positive or negative, respectively, as a categorical variable. Sequences were manually reviewed to verify classification of herpesvirus and polyomavirus sequences.

### 2.3 EBV variant analysis

For samples that were positive for EBV sequences, sequences were aligned to canonical EBV‐1 and EBV‐2 reference genomes (NCBI Reference Sequence: NC_007605.1 and NC_009334.1). The depth and breadth of coverage were calculated using RefCov (http://gmt.genome.wustl.edu/packages/refcov/), and alignments were reviewed to determine EBV type. Samples in which >70% of the EBV‐1 genome represented in the sequencing data were included in subsequent comparative analysis. Variants compared to the reference EBV‐1 genome were identified using Varscan, a platform‐independent software tool developed at the McDonnell Genome Institute at Washington University to detect variants in genomic data.^24^ For variant analysis, nucleotide positions with <10× read depth were classified as unevaluable and excluded. Variants and coverage were manually reviewed using Tablet, a high‐performance graphical viewer for metagenomic sequence assemblies and alignments.^25^

### 2.4 Data submission

Submission of microbial sequencing data to the public Sequence Read Archive is in progress at the time of submission, and BioProject and SRS identifiers will be provided prior to publication.

#### 2.4.1 Polymerase chain reaction

Tissue specimens were tested by quantitative PCR assays for EBV and the predominant anellovirus species in humans, torque teno virus (TTV), alpha subtype. Details of the PCR assays are described in the Supplementary Methods.

#### 2.4.2 Statistical analyses

Details of the statistical analyses are in the Supplementary Methods.

## 3 RESULTS

### 3.1 Subjects and samples

We identified 163 records in the Department of Pathology tissue archives, of which 69 specimens from 60 subjects from the period 1991‐2015 were adequate for analysis according to the described criteria. The male: female ratio was 36:24. The median age of the subjects was 15.1 years (range 0.1 to 67.9 years; mean 22.7, <21 years at time of transplant = 35/60). Subjects with polymorphic PTLD were younger at transplant (median age 13.0 years) than other groups, while the subjects with T cell PTLD were older at transplant (median age 40.7 years) than other groups (Table 1).

**Table 1:** Study subject (n = 60) and sample (n = 69) characteristics

	Polymorphic	DLBCL	Burkitt/Plasma Cell	T cell	Classic Hodgkin	Total
Patients	16	30	3	5	6	60
Age at transplant (years ± SD)	12.98 ± 17.16	27.78 ± 22.90	15.23 ± 25.78	40.66 ± 23.43	28.57 ± 17.35	
Age at transplant under 21 years	14 (87.5)	16 (53.33)	2 (66.67)	1 (20)	2 (33.33)	35
Male	11 (68.75)	16 (53.33)	2 (66.67)	3 (60)	4 (66.67)	36
Subject death^a^	6 (37.5)	14 (46.67)	1 (33.33)	4 (80)	1 (16.67)	26
Death‐censored graft failure^a^	2 (12.5)	2 (6.67)	0 (0)	0 (0)	1 (16.67)	5
Organ transplant type	16	30	3	5	6	60
Kidney	3 (18.75)	5 (16.67)	0 (0)	3 (60)	4 (66.67)	15
Liver	0 (0)	5 (16.67)	0 (0)	0 (0)	0 (0)	5
Heart	2 (12.5)	6 (20)	1 (33.33)	0 (0)	0 (0)	9
Lung	9 (56.25)	11 (36.67)	2 (66.67)	2 (40)	1 (16.67)	25
Bone marrow	1 (6.25)	0 (0)	0 (0)	0 (0)	1 (16.67)	2
Multiple	1 (6.25)	3 (10)	0 (0)	0 (0)	0 (0)	4
Median months from transplant to PTLD (Q1, Q3)	37 (6, 69)	28.5 (7, 127)	97 (85, 145)	132 (57, 164)	116 (102, 199)	
Sample location	18	34	5	6	6	69
Lymph node	7 (38.89)	4 (11.76)	1 (20)	1 (16.67)	6 (100)	19
GI tract	1 (5.56)	14 (41.18)	5 (100)	1 (16.67)	0 (0)	21
Liver	1 (5.56)	5 (14.71)	0 (0)	0 (0)	0 (0)	6
CNS	0 (0)	2 (5.88)	0 (0)	1 (16.67)	0 (0)	3
Disseminated	1 (5.56)	3 (8.82)	1 (20)	3 (50)	1 (16.67)	9
Bone Marrow	1 (5.56)	0 (0)	0 (0)	2 (33.33)	0 (0)	3
Lung	2 (11.11)	6 (17.65)	0 (0)	0 (0)	0 (0)	8
Other	7 (38.89)	6 (17.65)	0 (0)	3 (50)	0 (14.29)	16

Numbers in parentheses indicate percentages. DLBCL, diffuse large B cell lymphoma.

a Subject death and death‐censored graft failure were assessed within 5 years of the diagnosis of PTLD.

The transplanted organ was distributed across all the major types, lung being the most common (Table 1). PTLD was located in diverse locations, lymph node and GI tract being the most common. Applying the World Health Organization (WHO) classification of PTLD,^26^, ^27^ our 69 tissue cases were mostly monomorphic B cell type in 39 (of which 34 were DLBCL and five were other types). For the 8 subjects who had more than 1 PTLD occurrence (one subject had two recurrences), the PTLD WHO type was the same in each occurrence, except in one subject, who had a Hodgkin type PTLD initially and a DLBCL type in the recurrence 3 years later.

Death‐censored graft failure occurred in 8/60 (six in patients who later died) at a median time of 3.2 years after PTLD diagnosis (IQR 0.6‐7.2 years). Of the eight graft failures, five grafts failed within 5 years of the PTLD diagnosis. Thirty‐two patients had died at the time the samples were analyzed, at a median time of 3.5 years after PTLD diagnosis (IQR 0.7‐8.1 years, range 0–18.8 years). Of the 32 deaths, 26 died within 5 years of the PTLD diagnosis.

### 3.2 Detection of viral nucleotide sequences by MSS

**Figure 1:**
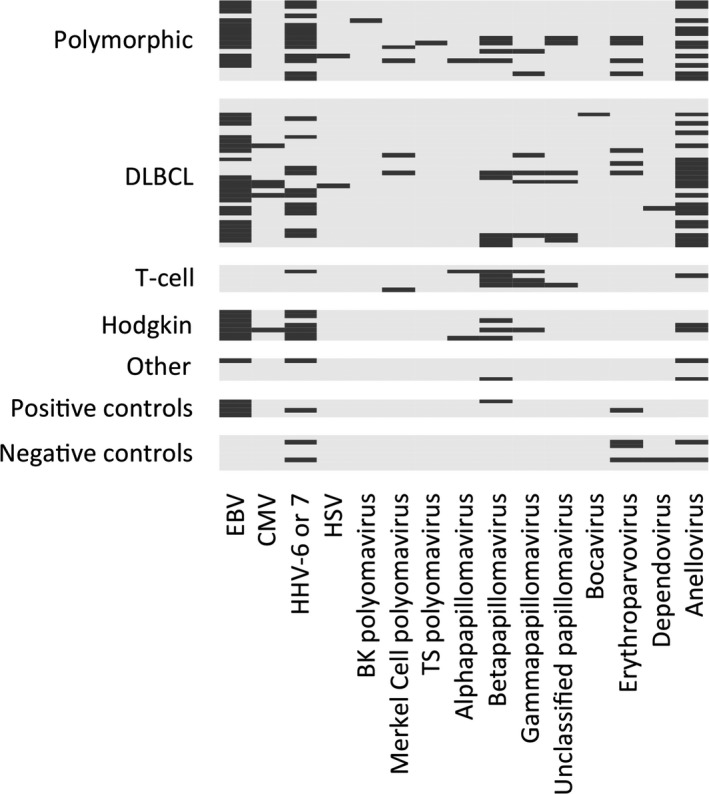
DNA viruses detected in stored PTLD tissues, stratified by WHO type. Each row represents a different sequenced sample. Viruses are noted in columns. A dark bar indicates the virus was detected in that sample, and gray background indicates the virus was not detected

Nucleotide sequences from at least one virus were detected by MSS in 86% (59/69) of the sequenced PTLD samples. Figure 1 depicts the viral sequences detected. EBV was detected in 61% (42/69) of the PTLD samples, and it was also detected in all of the 4 EBV positive control samples but not in any of the eight negative controls. Of the single‐stranded DNA viruses, anelloviruses were detected in 52% of the PTLD samples. Positivity by MSS for a viral genus was handled as a categorical yes/no variable in further analyses. When correlating with WHO classification, all six Hodgkin lymphoma cases were EBV sequencing positive and 5/6 were roseolovirus positive while all six T cell PTLDs were EBV sequencing negative (see Figure 1 for breakdown according to WHO PTLD type and see Figure S1). Anellovirus positivity by MSS did not associate with PTLD WHO type (Figure S1).

### 3.3 EBV genome variants

**Figure 2:**
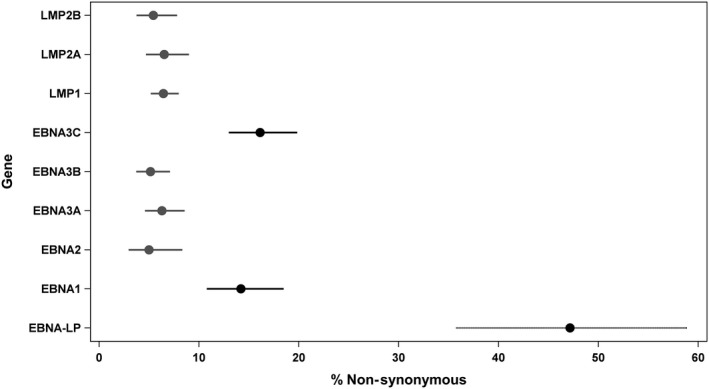
Plots of adjusted percent nonsynonymous sequence changes (nonsynonymous*100/nonsynonymous + synonymous + no change) in **EBV** genes, with 95% confidence intervals, as calculated by logistic regression analysis that included a random effect to adjust for repeated measurements. Differently colored and/or patterned lines correspond to significantly different genes (Tukey adjustment)

We had >70% of the EBV genome length represented in the sequence data from samples from 37 unique PTLD patients, 33 EBV‐1 and 4 EBV‐2. The 33 EBV‐1 samples were used for subsequent comparative analysis. We chose to focus on nine specific EBV genes that are most associated with oncogenesis or with viral latency profiles.^2^ We determined the nucleotide variants in each of these nine genes and classified variants as synonymous (no change in the predicted amino acid coding) vs nonsynonymous (predicted change in the amino acid coding, and therefore more likely to be pathogenic). As shown in Table 2, the genes *EBNA3C, EBNA‐LP,* and *LMP1* had a greater ratio of nonsynonymous changes to synonymous changes, suggesting a greater chance of pathogenic variants within these genes. Logistic regression analyses showed that the genes with the highest percent nonsynonymous changes were *EBNA‐LP* (significantly higher than all other genes), and *EBNA1* and *EBNA3C* (significantly lower than *EBNA‐LP* but significantly higher than all other genes; Figure 2).

**Table 2:** Changes found in nine Epstein‐Barr virus genes in 33 PTLD tissues, compared to the reference EBV‐1 genome. Bolded numbers represent genes where the percentage of nonsynonymous changes (change in coded amino acid) was significantly higher than in other genes evaluated (see also Figure 2)

Gene	Total nucleotide positions in gene^a^	Variant nucleotide positions^c^ (percentage of total positions)^c^	Total variant positions compared in 33 tissues^d^^,^ e	Evaluable nucleotide positions^e^	Positions with no change (% of evaluable)	No. of synonymous changes (% of evaluable)	No. of nonsynonymous changes (% of evaluable)
EBNA1	1926	23 (1.19)	759	553	379 (68.5)	89 (16.8)	85 (**15.4**)
EBNA2	1464	10 (0.68)	330	278	241 (86.7)	20 (7.2)	17 (6.1)
EBNA3A	2835	31 (1.09)	1023	877	764 (87.1)	47 (5.4)	66 (7.5)
EBNA3B	2817	35 (1.24)	1155	967	851 (88.0)	57 (5.9)	59 (6.1)
EBNA3C	2979	46 (1.54)	1518	1305	959 (73.4)	117 (9.0)	229 (**17.5**)
EBNA‐LP	1521	3 (0.2)	99	89	42 (47.1)	5 (5.6)	42 (**47.2**)
LMP1	1161	220 (17.23)	6600	5629	5099 (90.6)	97 (1.7)	433 (7.7)
LMP2A	1493	25 (1.67)	825	727	614 (98.0)	57 (7.8)	56 (7.7)
LMP2B	1137	21 (1.85)	693	617	523 (84.8)	54 (8.8)	40 (6.5)

a Total nucleotide positions in the coding sequence of each gene.

A position is considered “variant” if the nucleotide defined by MSS in one or more of the 33 samples analyzed differs from the reference nucleotide at that position.

b Percentage of coding sequence nucleotide positions for which a variant from the reference sequence is shown to be present in one or more samples.

c Total subjects’ nucleotide positions of change for each gene. For example, EBNA1, 759 equals 23 nucleotide positions of change × 33 specimens.

d Total positions compared = # of synonymous changes + # of nonsynonymous changes + # no change + # positions without enough coverage to evaluate.

e A nucleotide position was considered evaluable in a specific sample if it was represented in 10 or more sequencing reads.

### 3.4 MSS viral genome detection in EBV‐negative PTLD

The distribution of the different viruses in the EBV‐negative PTLD tissues is shown in Figure 1 and is further described in Supplementary Results.

### 3.5 Comparison of EBV and anellovirus detection methods

We confirmed that our tissue MSS results for EBV and anellovirus by quantitative tissue PCR. TTV levels by PCR were significantly higher in samples that were positive for anellovirus by MSS, compared to those that were negative by MSS (*P* < 0.001, data not shown). Copy loads for EBV and TTV by PCR showed a modest but significant association with each other (Pearson correlation coefficient rho = 0.36, *P* < 0.001, data not shown).

### 3.6 Survival analysis (patient death)

#### 3.6.1 Contingency analyses

We first analyzed for survival outcomes in relation to MSS or qPCR results by using contingency analyses (Table S1). Patient death within 5 years of PTLD diagnosis was associated with anellovirus (30% dead if negative vs 57% dead if positive; *P* = 0.037) and gammapapillomavirus (38% dead if negative and 86% dead if positive; *P* = 0.036) positivity by MSS. Death was not associated with EBV positivity (any method–clinical tumor positivity, MSS or PCR positivity), WHO classification type or early vs late onset PTLD. The only clinical parameter to associate with higher patient death in contingency analyses was liver location of PTLD (*P* = 0.031). Anellovirus tissue MSS positivity did not associate with PTLD WHO type, any specific location of PTLD or age at transplant.

#### 3.6.2 Tissue qPCR

**Figure 3:**
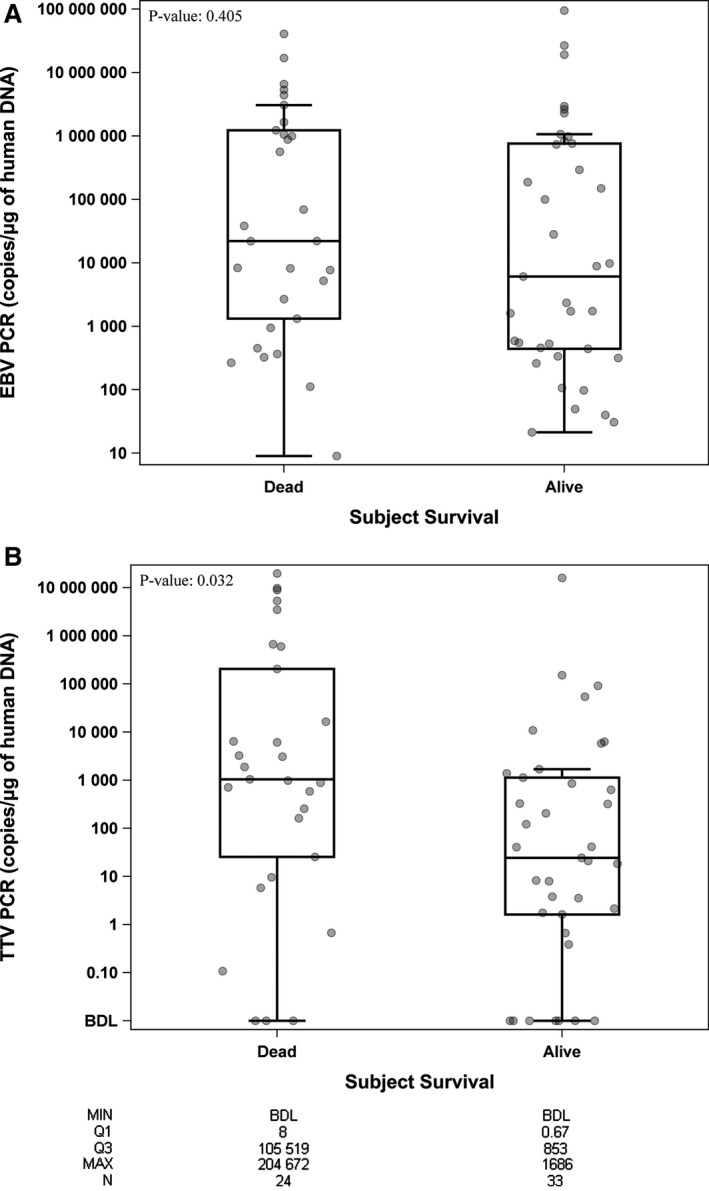
Results of EBV or TTV quantitative PCR vs patient survival after PTLD. (A) Box and whiskers plot of EBV tissue PCR (copies/μg human DNA), stratified by patient alive or dead at 5 years after PTLD diagnosis (*P* = NS). (B) Box and whisker plot of TTV tissue PCR (copies/μg human DNA), where higher TTV qPCR loads were present in patients dead at 5 years after PTLD diagnosis (*P* = 0.032). The *Y*‐axis is shown on log base 10 scale. Values below detection limit were assigned a value of 0.00001. Data points correspond to the SAMPLES (there can be >1/subject), and are “jittered” so that samples with overlapping markers are separated from each other. Box‐plot characteristics: Line = Median, Top edge of box = 75th percentile (Q3), Bottom edge of box = 25th percentile (Q1), Upper and lower whiskers = 1.5 × IQR (Q3‐Q1)

We also analyzed the relationship between patient presentation or survival and levels of EBV and TTV measured by qPCR. Neither EBV PCR copy number nor TTV PCR copy number was associated with PTLD WHO type (data not shown). EBV PCR copy load did not associate with patient death (Figure 3A). In contrast, TTV PCR loads were significantly higher in patients who died (*P* = 0.032; Figure 3B). The median TTV copy number was 122 in the overall cohort and was 931 in patients who died within 5 years of PTLD diagnosis (vs 21 in those still alive at 5 years). Patient death occurred in 72% of those with TTV tissue load above the median of 122, whereas patient death was only 43% for those with TTV loads below the median (*P* = 0.024).

#### 3.6.3 Time to event analyses

We then analyzed covariate associations to patient death within 5 years of PTLD diagnosis using time‐to‐event analyses (Table 3). Univariable Cox regression analyses revealed that, liver, CNS or bone marrow locations of PTLD were significantly associated with worse patient survival. MSS positivity for anelloviridae (HR 2.00, 95% CI 0.09, 4.70, *P* = 0.09) and gammapapillomavirus (HR 2.38, 95% CI 0.87, 5.62, *P* = 0.06) trended toward worse patient survival. In subset sensitivity analyses where T cell PTLD was excluded, anellovirus positivity by MSS was significantly associated with patient death within 5 years of PTLD diagnosis (Supplementary Results).

**Table 3:** Univariable Cox regression analysis of relationship of covariates with patient death within 5 years of PTLD diagnosis

Variable	Reference group	Hazard Ratio (95% CI)	*P*‐value
Age at transplant (Years)	Each 1 year age increment	1.01 (0.99, 1.03)	0.18
Male	Female	0.97 (0.45,2.17)	0.94
Transplant type			
Heart	Bone marrow	0.33 (0.04,6.86)	0.85
Liver	Bone marrow	0.78 (0.10,15.98)	
Lung	Bone marrow	0.55 (0.10,10.15)	
Multi‐organ	Bone marrow	0.58 (0.05,12.71)	
Kidney	Bone marrow	0.40 (0.06,7.69)	
Locations of PTLD			
Lymph node	Non‐lymph node location	0.45 (0.15,1.09)	0.10
GI tract	Non‐GI tract location	0.87 (0.34,1.97)	0.74
**Liver**	**Non‐liver location**	**4.15 (1.20,11.17)**	**0.005**
**CNS**	**Non‐CNS location**	**6.74 (0.36,36.73)**	**0.037**
**Bone Marrow**	**Non‐bone marrow location**	**14.74 (2.99,61.04)**	**<.001**
Lung	Non‐lung location	0.43 (0.07,1.47)	0.24
Time from transplant to PTLD			
1 year or above	<1 year	0.81 (0.37,1.90)	0.61
PTLD type			
Polymorphic	Non‐polymorphic	0.66 (0.24,1.54)	0.36
DLBCL	Non‐DLBCL	1.25 (0.58,2.76)	0.57
Classic Hodgkin	Non‐Hodgkin	0.35 (0.02,1.64)	0.28
**T cell**	**Non‐T cell**	**3.58 (1.04,9.40)**	**0.013**
Burkitt lymphoma/plasma cell	Non‐Burkitt/Plasma	0.74 (0.04,3.48)	0.76
Induction immunosuppression Regimen^ other	Anti‐thymocyte globulin	0.87 (0.29,2.87)	0.22
Unknown	Anti‐thymocyte globulin	0.55 (0.15,1.97)	
Anti‐IL2 receptor	Anti‐thymocyte globulin	1.74 (0.56,5.89)	
**Log TTV copies/μg of Human DNA** *	**Each 1 log increase**	**1.10 (1.02,1.20)**	**0.016**
**TTV‐ copies/μg of Human DNA: >median** *	**TTV‐1 copies/μg of Human DNA: ≤ median**	**2.23 (0.98,5.51)**	**0.058**
Tumor EBV status (clinical testing) Any Positive@	Negative	0.55 (0.25,1.27)	0.14
Log EBV copies/μg of Human DNA*	Each 1 log increase	1.01 (0.93, 1.11)	0.76
EBV copies/μg of Human DNA: > median*	EBV copies/μg of Human DNA: ≤ median	1.28 (0.57,2.97)	0.55
MSS positive			
EBV	Negative	0.67 (0.31,1.47)	0.30
Cytomegalovirus	Negative	0.52 (0.03,2.47)	0.51
HHV6 or 7	Negative	1.15 (0.52,2.49)	0.73
Simplexvirus	Negative	2.45 (0.14,11.84)	0.37
BK_polyomavirus	Negative	4.76 (0.26,24.46)	0.10
Merkel_cell_polyoma	Negative	0.52 (0.03,2.48)	0.52
Alphapapillomavirus	Negative	1.05 (0.06,4.94)	0.97
Betapapillomavirus	Negative	1.75 (0.68,4.00)	0.20
**Gammapapillomavirus**	**Negative**	**2.38 (0.87,5.62)**	**0.06**
Papillomaviridae	Negative	1.85 (0.44,5.32)	0.31
Erythroparvovirus	Negative	0.76 (0.12,2.54)	0.70
**Anelloviridae**	**Negative**	**2.00 (0.91,4.70)**	**0.09**

Number dead within 5 years = 26 and number alive at 5 years post‐PTLD = 34, except where marked separately.

^^^Number dead = 25, number alive = 34, *Number dead = 24, number alive = 33, ^@^Number dead = 25, number alive = 30.

For log‐transformed PCR values, 1 was added to all values to account for PCR copy number = 0, as log 0 is not defined and log 1 = 0.

PTLD locations were not mutually exclusive.

A quantified TTV PCR load greater than the median of 122 copies/μg human DNA also trended with higher patient death (HR 2.23, 95% CI 0.98, 5.51, *P* = 0.058). Notably, the log‐transformed TTV viral load was strongly associated with patient death (hazard ratio 1.10, 95% confidence intervals 1.02, 1.20, *P* = 0.016), suggesting that higher tissue anellovirus quantity has a dose‐response relationship with patient death.

In contrast, EBV positivity by any method, including log‐transformed copy number, did not associate with patient death in time‐to‐event (Table 3) or sensitivity analyses (Supplementary Results).

#### 3.6.4 Multivariable analyses

Finally, based on our univariate associations and known literature, we fitted a multivariate Cox regression model where 4 variables were considered for inclusion: age at transplant, T cell PTLD, log‐transformed EBV PCR copy number, log‐transformed TTV PCR copy number. Only T cell type PTLD (adjusted hazard ratio 4.14; 95% confidence intervals 1.18, 11.28, *P* = 0.006) and log‐transformed TTV PCR copy number (adjusted hazard ratio 1.11; 95% confidence intervals 1.02, 1.20, *P* = 0.012) remained independently significant.

## 4 DISCUSSION

In this study, we were able to successfully recover multiple DNA viral genomes from stored FFPE tissue, identify viruses in sequence data, and assess for sequence variants in key EBV genes. A key strength of our study was the broad range of viruses detectable, made possible by using a sequencing approach rather than a targeted PCR‐based approach. We are able to achieve a high degree of coverage of the EBV genome and detected many variants, across all the nine EBV genes tested. Certain EBV genes had higher percentages or proportions of nonsynonymous nucleotide variants.

Data on the genomic diversity of EBV and their contribution to PTLD pathogenesis or outcomes are scant, with small sample sizes in all, given the rarity of this disease. Vaysberg et al, from a panel of five EBV+ B cell lymphomas, identified three distinct and different variants of *LMP1*, with 2 gain of function mutations, which induced sustained MAP kinase activation and c‐Fos induction.^28^ Notably, we detected one of these gain of function mutations, S366T, in three of our polymorphic PTLD samples. Using FFPE tissues, Nourse et al found that EBV‐miRNA was profiled reliably within archival FFPE tissue in 14/23 patients, but not in tissues with low abundance of EBV.^29^ In subsequent studies, the same group found that nine CNS and 16 systemic PTLD tissues expressed similar viral latent (*EBNA2, EBNA3A, LMP1*) and lytic (*BZLF1, BRLF1, BLLF1*) gene mRNA transcripts.^30^

From studies of other EBV‐associated cancers in a nontransplant setting in a general population, the genes *BRLF1*,* BBRF3*, and *BBLF2/BBLF3* had significant associations with gastric carcinoma.^31^ In Argentina, investigators detected an association between specific *BZLF1* gene variants such as *BZLF1‐A2* with lymphomas and *BZLF1‐C* with infectious mononucleosis.^32^ Specific polymorphisms in two viral gene promoters Cp and Qp were found in nasopharyngeal carcinoma.^33^ A clonal *LMP1* gene containing a 30 bp deletion (del30) was found in 46.1% of NK/T cell lymphomas and only in 4.8% of the controls, with much worse patient survival in those with this deletion.^34^ However, as shown in endemic Burkitt lymphoma, within a geographic region, different EBV genetic variations can coexist,^35^ such that specific gene variation associations with presentation or prognosis have been difficult. Although our study was not powered to detect individual variants associated with survival or PTLD type, we have demonstrated the technology can be successfully be used for this approach. The significance of specific EBV variants should be explored in future multicenter studies.

Prior studies of EBV‐negative DLBCL PTLD cases (n = 9) have shown that *human* gene alterations in these cases are more similar to those seen in immunocompetent Hodgkin lymphoma or non‐Hodgkin lymphoma, rather than the human gene alterations seen in EBV‐positive PTLDs (n = 24).^36^, ^37^ These results suggested that viral oncogenesis is not a key pathological pathway in EBV‐negative DLBCL PTLD. Our results, where no DNA viral genus was overrepresented in EBV‐negative PTLD tissue samples, would support this hypothesis, though our larger sample size of 27 EBV‐negative PTLDs is also relatively small.

While we are not aware of any reported association between gammapapillomavirus and degree of overall immunosuppression, the association of anellovirus positivity with patient death in our exploratory analyses was of particular interest. The biological significance of the entire anellovirus group is unknown and evolving.^38^, ^39^ Using cell‐free DNA sequencing from plasma samples derived from thoracic organ transplant recipients, De Vlaminck et al^40^ found a marked expansion of the annelloviridae family upon the onset of immunosuppression and a lower AnV burden with acute rejection episodes, even with appropriate drug immunosuppression levels. Blatter et al found that low AnV loads in pediatric lung transplant recipients at 2 weeks posttransplantation were more likely to develop acute rejection within 3 months after transplant (*P* = 0.013).^41^ High Anv loads from broncholaveolar lavage samples in lung transplant patients correlated with dysbiotic bacterial communities in the allograft. 42 In adult kidney transplantation, low levels of AnV associated with higher risk of late acute antibody‐mediated rejection.^43^ These findings suggest that anelloviruses can be a biomarker for the overall degree of immunosuppression achieved.^40^

Strengths of our study include demonstrating the feasibility of MSS of old, stored FFPE tissue for detection of *viral* genomes, the use of actual human PTLD specimens rather than in vitro cell lines to investigate for EBV variants, and the possible association with clinical presentations and outcomes. Limitations of the study include its single center and retrospective nature. Determining which microbial gene variants are pathogenic is more challenging than with human gene variants. In the latter, many resources are available to help determine pathogenicity, such as well‐annotated reference genomes, published literature on mutants that are associated with clinical pathology, and prediction models. Such tools are not as well‐developed for most microbial genome variants. An additional limitation of the MSS technology is its analysis of samples for short genomic reads, which may miss larger deletions. The fragmented nature of nucleic acid from FFPE tissue also compounds the difficulty in distinguishing true deletions from missing sequence coverage. For our MSS results, we emphasized specificity over sensitivity; our strictness in calling a sample positive may have been too stringent. EBV is the driver for PTLD onset,^2^, ^44^, ^45^, ^46^ thus other agents may act in the modulation of disease progression. Finally, patient survival can be very different in different organ transplants with different locations or WHO types of PTLD. We have accounted for WHO type; T cell PTLD remained independently significant in multivariate models, consistent with other recent reports.^47^ Certain individual locations were associated with higher risk of patient death in univariate analyses in our study, but each was present in very few subjects. Individual organ transplant type was not a significant univariate predictor in our study population. Notably, the prior PTLD‐1 trial evaluated survival after a common treatment regimen across different organ transplants and different WHO types. A completely homogenous PTLD study population is not possible given the relative rarity of this disease. Immunosuppression regimen associations were difficult to assess given the long time period when samples were acquired and the variety of regimens across organs.

In future, we expect to characterize further the specific types of EBV variants (single nucleotide variants, insertions, deletions, missense mutations, etc.) in the nine EBV genes we have analyzed so far. We will also expand our analyses to other EBV genes, using a larger cohort for greater power. Future studies could also involve laser capture of single malignant PTLD cells from tissue, and single cell RNA sequencing, but our study is the necessary first step.

## CONFLICT OF INTEREST

None relevant to this work.
