# Supplementary material for: Metagenomic analysis of DNA viruses from posttransplant lymphoproliferative disorders
Source: Cancer Med. 2019 Jan 29;8(3):1013–23. doi: 10.1002/cam4.1985 (PMC6434222; doi:10.1002/cam4.1985)
Supplement: Supplementary file 1 [file CAM4-8-1013-s001.docx]

**Supplementary Methods**

*DNA extraction*

Tissue core punches (1.2mm) or scrolls (10µm) were harvested from paraffin blocks and submitted for DNA extraction following a 3-step process of deparaffinization, enzymatic digestion, and silica column purification using the QIAamp® DNA micro Kit (Qiagen). The concentration of the resultant DNA was determined via the Qubit® 2.0 Fluorometer using either the Qubit™ dsDNA HS Assay Kit or Qubit™ dsDNA BR Assay Kit.

*Pathology covariates*:

From the Pathology database the following data elements were extracted and reviewed for accuracy by re-examining the tissue: 1) Final diagnosis/pathological classification (PTLD, polymorphic/monomorphic, further classification of lymphoma subtype in cases of monomorphic PTLD); 2) Lineage determination markers (B cell, T/NK, plasmacytic), presence of EBV (*EBER* *in situ* hybridization in most cases, or immunohistochemistry for the EBV latent membrane protein 1 (*LMP1*) or EB nuclear antigen 1 (*EBNA1*) in some cases); 3) Ancillary test results including data from flow cytometry, molecular and cytogenetic analysis when available.

*Clinical covariates***:**

Clinical data elements extracted from the electronic medical record included: 1) Recipient demographics (age at transplant, sex, race, age at PTLD); 2) Recipient and donor serostatus (EBV and CMV); 3) Recipient transplant characteristics (type of organ/hematopoietic transplant, date of and age at transplant, induction/maintenance immunosuppression); 4) Donor type (related/unrelated, deceased or living, standard/extended/cardiac death, HLA matching to recipient); 5) PTLD date of diagnosis; 6) location(s) of PTLD; 7) PTLD outcomes (complete versus partial remission, progression, recurrence); 8) patient death or death-censored graft failure after PTLD.

*Polymerase chain reaction*

To quantify EBV in the specimens a laboratory-developed quantitative Taqman real-time PCR assay was employed. The primers and TaqMan probe used are contained within an amplicon originally described by Wandinger et al ^43^. The lab used Primer Express to design a set of TaqMan primers and a TaqMan MGB probe based on the amplicon described in this paper. The sequences detected by these primers and probe are found within the EBV *EBNA1* gene.

Each PCR reaction was performed in a final reaction volume of 50µl using ABI Taqman Universal PCR master mix, including 5µl of specimen nucleic acid extract, 0.9 µM forward primer (EBV-F 5' GGTAGTAAGACCTCCCTTTACAACCTAA 3'), 0.9 µM reverse primer (EBV-R 5' TGTAAGACGACATTGTGGAATAGCA 3'), and 0.25 µM for the probe (EBV-MGB 5' 6FAM-CGAGGAACTGCCC-MGBNFQ 3'). The reaction was run in an Applied Biosystems 7500 Real-Time PCR System instrument with a program of: 50°C for 1min; 95°C for 10 min; 40 cycles of 95°C for 15 sec and 60°C 1 min. A set of standards, consisting of a series of six 5-fold dilutions of a commercial quantitated DNA standard (Advanced Biotechnologies Inc. Cat. # 08-925-000, Eldersburg, MD) was included in each run.

To quantify TTV, TaqMan quantitative real-time PCR was performed, using an amplicon that was previously described ^44^. The TTV PCR assay targets a highly conserved segment of the viral untranslated region. Each TTV reaction was performed in 25 μL total volume, including 5 μL extracted specimen, ABI TaqMan Universal PCR Master Mix (Applied Biosystems), 0.9 μM forward primer (5′ TGCCGAAGGTGAGTTTACACA 3′), 0.9 μM reverse primer (5′ TTCAGAGCCTTGCCCATAGC 3′), and 0.25 µM probe (5′ 6FAM-CCCGAATTGCCCCTTGAC-MGBNFQ 3'). Cycling was carried out on the ABI 7500 instrument with the following conditions: 50°C for 2 minutes and 95°C for 10 minutes, followed by 45 cycles of 95°C for 15 seconds and 60°C for 1 minute. The quantitation standard consisted of a synthesized 144 bp region from the conserved, untranslated TTV genome, which contained the target for PCR, inserted into the pUC57 plasmid. 10-fold dilutions of the plasmid, starting with 10^8^ copies per ml were used for quantitative standards.

Human DNA in each specimen was quantified using the Applied Biosystems Quantifiler Human DNA Quantification Kit (Cat No. 343895; Foster City, CA). EBV and TTV levels were expressed as copy number per microgram of human DNA.

*Statistical analyses*:

Univariable associations between the outcomes (patient death or death-censored graft failure within 5 years of first PTLD diagnosis) and covariates were assessed using a chi-square test or Fisher’s exact test as appropriate. Time-to-event univariable analyses for association with patient death within 5 years of PTLD diagnoses were conducted by Cox regression. A log transformation (+1) was applied to covariates with strongly right-skewed distributions. To test for associations between covariates and time-to-event outcomes, score tests were used, and 95% confidence intervals for hazard ratios were estimated using the profile likelihood approach. Key variables of interest (age at transplant, T cell PTLD, EBV PCR level and TTV PCR level) were considered for inclusion in multivariable Cox-regression models, and significant variables were retained in the final model. Pearson correlation, Wilcoxon-rank sum, and Kruskal-Wallis tests were used in the analysis of genome detection techniques. Association of proportions of non-synonymous variants between EBV genes was performed by a logistic regression, adjusted for correlation of repeated measures on the same subject (generalized linear mixed model). Tukey’s adjustment for multiple comparisons was used to test for differences in the percent non-synonymous changes among genes.

*In patients with more than one PTLD event, only the covariates associated to the first PTLD event/sample (collected prior to any treatment) were considered in the statistical analyses.* All analyses were carried out using SAS version 9.4 (Cary, NC). A p < 0.05 was considered as significant.

**Supplementary Results**

*MSS viral genome detection in EBV-negative PTLD*

In the EBV-negative PTLD tissue samples, we compared the frequency of MSS positivity for each DNA virus to the relative frequency of that virus in EBV-positive PTLD samples. No viruses were present in higher proportion in the 27 EBV-negative PTLD tissues, in comparison to the 42 EBV-positive PTLD tissue samples. The distribution of the different viruses found by MSS in the EBV negative PTLD tissues is shown in Figure 1 of the main paper.

*Sensitivity analyses*

Since the 5 T cell PTLD cases were all EBV negative by clinical testing/MSS and yet had 80% mortality within 5 years of PTLD diagnosis, we performed an additional sensitivity analysis of patient death by covariates on the 55 subjects excluding the 5 T cell PTLD cases. In this analysis, EBV positivity by clinical testing still did not associate with patient death, but anellovirus positivity became significant (HR 2.63, 95% CI 1.08, 7.35, p=0.036).

We did not find any significant association between tissue anellovirus MSS positivity and the initial use of tacrolimus, cyclosporine, mycophenolate, azathioprine or steroids.

*Association of patient death within 5 years of PTLD diagnosis, to EBV gene variants, or to EBV MSS positivity by age group:*

Exploratory analyses in each gene showed that no individual non-synonymous variant was associated with either patient death within 5 years of PTLD diagnosis or with PTLD WHO type, though these exploratory analyses were not powered to detect significant differences. A previously characterized 30 bp *LMP1* deletion seen in non-transplant NK/T cell lymphomas^33^ was found in 6 of our cases but was not associated with patient death after PTLD (p=0.2).

We further analyzed for EBV positivity and patient survival using contingency table analysis at 5 years post-PTLD diagnosis, stratified by patient age groups, either at time of transplant or at time of PTLD diagnosis. We found a significant result for one group only: tissue EBV negativity by MSS was associated with higher risk of death, if age at PTLD diagnosis was > 18 years. In contrast, the survival did not significantly differ by EBV MSS positivity or negativity in other stratifications of age at diagnosis, and in none when stratified by age at transplant.

*Associations of covariates to death-censored graft failure:*

In contingency table analyses, death-censored graft failure within 5 years of PTLD diagnosis was not associated with covariates, including any viral genus by MSS, TTV copy number, EBV positivity (any method), with WHO classification, or time to PTLD. However, the number of graft failure cases was small, only 8 overall and 6 within 5 years of PTLD diagnosis.

**Supplementary figure legends**:

Figure 1

Panel A: Box and whiskers plot of EBV tissue PCR (copies/µg human DNA), stratified by PTLD WHO type (p = NS).

Panel B: Box and whiskers plot of TTV tissue PCR (copies/µg human DNA), stratified by PTLD WHO type (p = NS).

**Supplementary figure 1** Panel A


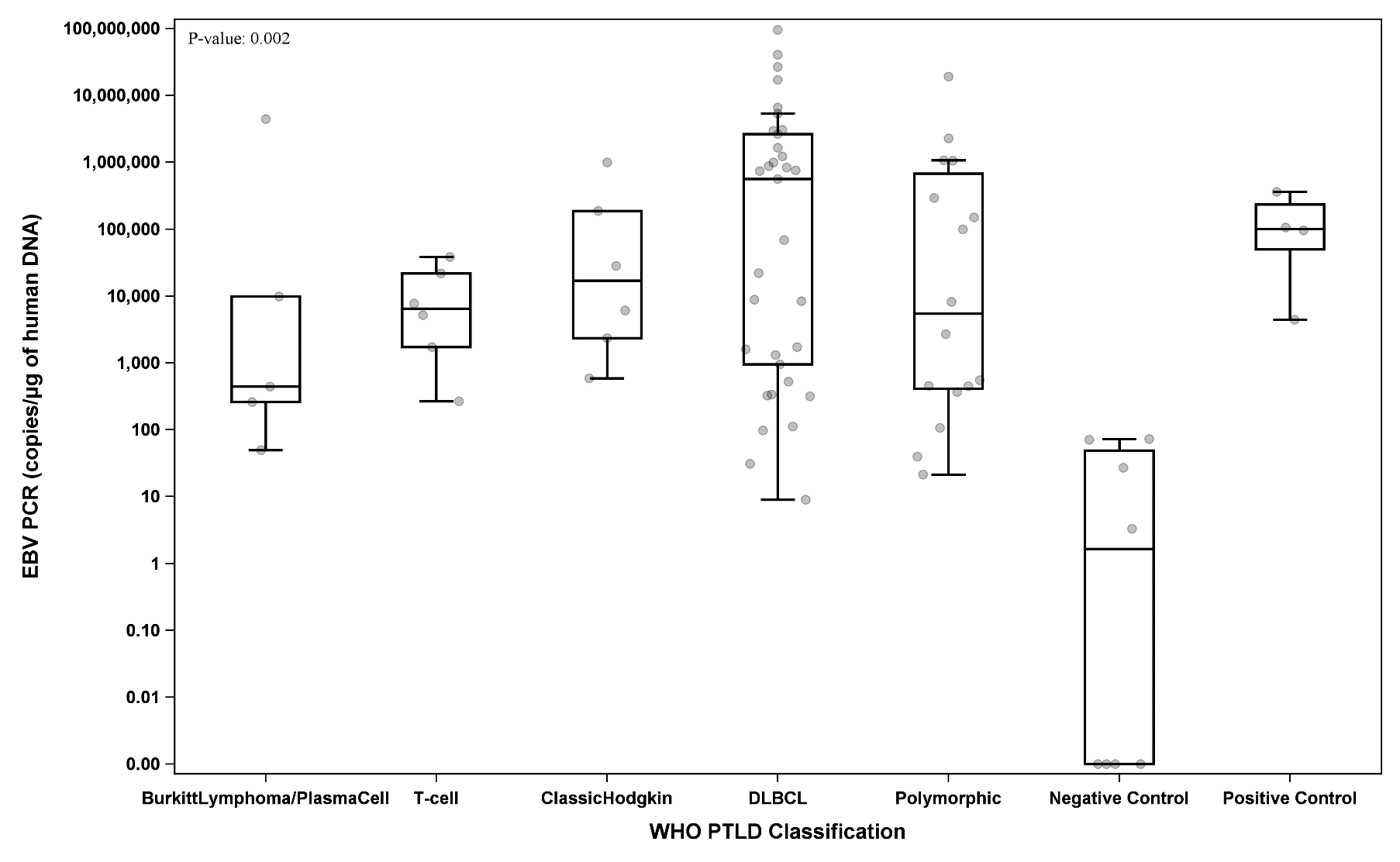


Panel B


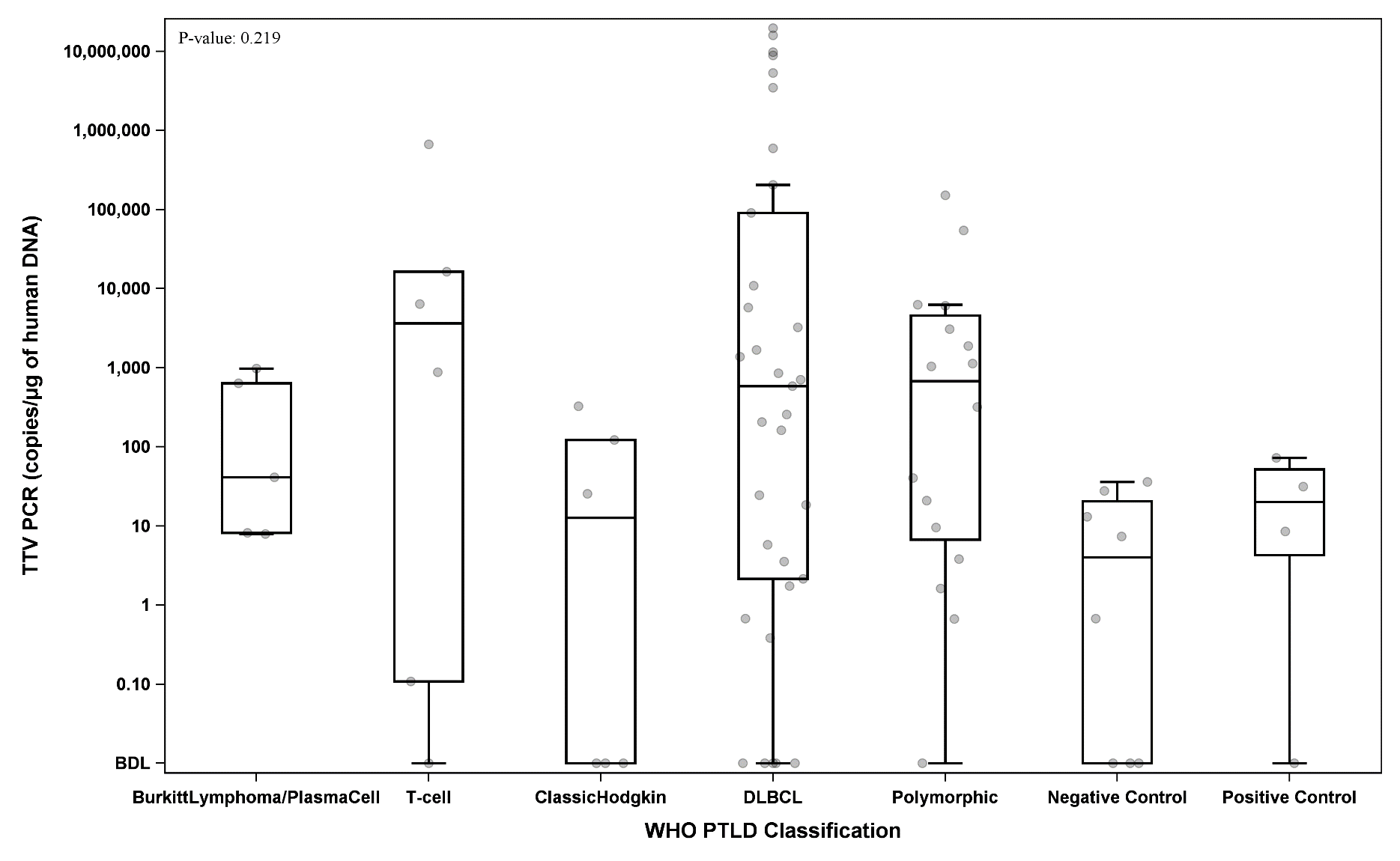


**Supplementary Table 1. Contingency table analyses univariable association of covariates with patient death, within 5 years of PTLD diagnosis**

| **Variable** | **Category** | **# surviving at 5 years (% of total in that row)** | **P-value** |
| --- | --- | --- | --- |
| Gender | Male | 21 (58) | 0.75* |
|  | Female | 13 (54) | . |
| Transplant Type | Renal/Kidney | 10 (67) | 0.87 |
|  | Lung | 13 (52) |  |
|  | Heart | 6 (67) |  |
|  | Bone Marrow | 1 (50) |  |
|  | Liver | 2 (40) |  |
|  | Multi-Organ | 2 (50) |  |
| Time from Transplant to PTLD | <1 Year | 8 (47) | 0.34* |
|  | 1 Year or Above | 26 (60) | . |
| Tumor Clinical EBV Status | Any Positive | 24 (62) | 0.10* |
|  | Negative | 6 (37) | . |
| PTLD WHO Classification | Polymorphic | 10 (63) | 0.58* |
|  | Non Polymorphic | 24 (55) | . |
|  | DLBCL | 18 (60) | 0.60* |
|  | Non DLBCL | 16 (53) | . |
|  | Classic Hodgkin | 5 (83) | 0.22 |
|  | Non Classic Hodgkin | 29 (54) | . |
|  | Monomorphic T-cell | 1 (20) | 0.16 |
|  | Non Monomorphic T-cell | 33 (60) | . |
|  | Burkitt/Plasma Cell | 2 (67) | 1.00 |
|  | Non Burkitt/Plasma Cell | 32 (56) | . |
| Induction Immunosuppression Regimen | Anti-thymocyte globulin | 7 (58) | 0.11 |
|  | Anti-IL2 receptor | 2 (22) | . |
|  | Other | 12 (60) | . |
|  | Unknown | 13 (72) | . |
| **Copies of TTV-1 per ug of Human DNA (Median)** | **Above 122.1 (Above Median)** | **12 (43)** | **0.0239*** |
|  | **≤ 122.1 (Below Median)** | **21 (72)** |  |
| Copies of EBV per ug of Human DNA (Median) | Above 7701.3 (Above Median) | 15 (52) | 0.34* |
|  | ≤ 7701.3 (Below Median) | 18 (64) |  |
| MSS - EBV | Non Positive (Count=0) | 11 (48) | 0.28* |
|  | Positive (Count>0) | 23 (62) |  |
| Cytomegalovirus | Non Positive | 32 (56) | 1.00 |
|  | Count>0 | 2 (66.67) | . |
| Roseolovirus | Non Positive | 18 (56) | 0.94* |
|  | Count>0 | 16 (57) | . |
| Simplexvirus | Non Positive | 34 (58) | 0.43 |
|  | Count>0 | 0 (0) | . |
| BK polyomavirus | Non Positive | 34 (58) | 0.43 |
|  | Count>0 | 0 (0) | . |
| Merkel cell polyoma | Non Positive | 31 (55) | 0.62 |
|  | Count>0 | 3 (75) | . |
| Trichodysplasia spinulosa-associated polyomavirus | Non Positive | 33 (56) | 1.00 |
|  | Count>0 | 1 (100) | . |
| Alphapapillomavirus | Non Positive | 32 (56) | 1.00 |
|  | Count>0 | 2 (67) | . |
| Betapapillomavirus | Non Positive | 29 (60) | 0.24* |
|  | Count>0 | 5 (42) | . |
| **Gammapapillomavirus** | **Non Positive** | **33 (62)** | **0.036** |
|  | **Count>0** | **1 (14)** | **.** |
| Papillomaviridae | Non Positive | 33 (59) | 0.31 |
|  | Count>0 | 1 (25) | . |
| Bocavirus | Non Positive | 33 (56) | 1.00 |
|  | Count>0 | 1 (100) | . |
| Erythroparvovirus | Non Positive | 31 (56) | 1.00 |
|  | Count>0 | 3 (60) | . |
| Dependoparvovirus | Non Positive | 33 (56) | 1.00 |
|  | Count>0 | 1 (100) | . |
| **Anelloviridae** | **0** | **21 (70)** | **0.037*** |
|  | **Count>0** | **13 (43)** | **.** |
| Lymph node location of PTLD | Present | 14 (74) | 0.07* |
|  | Not present | 20 (49) | . |
| GI tract location of PTLD | Present | 10 (59) | 0.83* |
|  | Not present | 24 (56) | . |
| **Liver location of PTLD** | **Present** | **0 (0)** | **0.031** |
|  | **Not present** | **34 (61)** | **.** |
| CNS location of PTLD | Present | 1 (50) | 1.00 |
|  | Not present | 33 (57) | . |
| Disseminated | Present | 3 (50) | 1.00 |
|  | Not present | 31 (57) | . |
| Bone Marrow | Present | 0 (0) | 0.08 |
|  | Not present | 34 (60) | . |
| Lung | Present | 6 (75) | 0.45 |
|  | Not present | 28 (54) | . |
